# Frontiers in antiviral therapy and immunotherapy

**DOI:** 10.1002/cti2.1115

**Published:** 2020-02-19

**Authors:** Steven M Heaton

**Affiliations:** ^1^ Department of Biochemistry & Molecular Biology Monash University 23 Innovation Walk Clayton VIC 3800 Australia

Globally, recent decades have witnessed a growing disjunction, a ‘Valley of Death’^1^, ^2^ no less, between broadening strides in fundamental biomedical research and their incommensurate reach into the clinic. Plumbing work on research funding and development pipelines through recent changes in the structure of government funding,^2^ new public and private joint ventures and specialist undergraduate and postgraduate courses now aim to incorporate pathways to translation at the earliest stages. Reflecting this shift, the number of biomedical research publications targeting ‘translational’ concepts has increased exponentially, up 1800% between 2003 and 2014^3^ and continuing to rise rapidly up to the present day. Fuelled by the availability of new research technologies, as well as changing disease, cost and other pressing issues of our time, further growth in this exciting space will undoubtedly continue. Despite recent advances in the therapeutic control of immune function and viral infection, current therapies are often challenging to develop, expensive to deploy and readily select for resistance‐conferring mutants. Shaped by the host‐virus immunological ‘arms race’ and tempered in the forge of deep time, the biodiversity of our world is increasingly being harnessed for new biotechnologies and therapeutics. Simultaneously, a shift towards host‐oriented antiviral therapies is currently underway. In this *Clinical & Translational Immunology* Special Feature, I illustrate a strategic vision integrating these themes to create new, effective, economical and robust antiviral therapies and immunotherapies, with both the realities and the opportunities afforded to researchers working in our changing world squarely in mind.

Opening this CTI Special Feature, I outline ways these issues may be solved by creatively leveraging the so‐called ‘strengths’ of viruses. Viral RNA polymerisation and reverse transcription enable resistance to treatment by conferring extraordinary genetic diversity. However, these exact processes ultimately restrict viral infectivity by strongly limiting virus genome sizes and their incorporation of new information. I coin this evolutionary dilemma the ‘information economy paradox’. Many viruses attempt to resolve this by manipulating multifunctional or multitasking host cell proteins (MMHPs), thereby maximising host subversion and viral infectivity at minimal informational cost.^4^ I argue this exposes an ‘Achilles Heel’ that may be safely targeted via host‐oriented therapies to impose devastating informational and fitness barriers on escape mutant selection. Furthermore, since MMHPs are often conserved targets within and between virus families, MMHP‐targeting therapies may exhibit both robust and broad‐spectrum antiviral efficacy. Achieving this through drug repurposing will break the vicious cycle of escalating therapeutic development costs and trivial escape mutant selection, both quickly and in multiple places. I also discuss alternative post‐translational and RNA‐based antiviral approaches, designer vaccines, immunotherapy and the emerging field of neo‐virology.^4^ I anticipate international efforts in these areas over the coming decade will enable the tapping of useful new biological functions and processes, methods for controlling infection, and the deployment of symbiotic or subclinical viruses in new therapies and biotechnologies that are so crucially needed.

Upon infection, pathogens stimulate expression of numerous host inflammatory factors that support recruitment and activation of immune cells. On the flip side, this same process also causes immunopathology when prolonged or deregulated.^5^ In their contribution to this Special Feature, Yoshinaga and Takeuchi review endogenous RNA‐binding proteins (RBPs) that post‐transcriptionally control expression of crucial inflammatory factors in various tissues and their potential therapeutic applications.^6^ These RBPs include tristetraprolin and AUF1, which promote degradation of AU‐rich element (ARE)‐containing mRNA; members of the Roquin and Regnase families, which respectively promote or effect degradation of mRNAs harbouring stem–loop structures; and the increasingly apparent role of the RNA methylation machinery in controlling inflammatory mRNA stability. These activities take place in various subcellular compartments and are differentially regulated during infection. In this way, mRNA‐destabilising RBPs constitute a ‘brake’ on the immune system, which may ultimately be toggled therapeutically. I anticipate continued efforts in this area will lead to new methods of regaining control over inflammation in autoimmunity, selectively enhancing immunity in immunotherapy, and modulating RNA synthesis and virus replication during infection.

Another mRNA under post‐transcriptional regulation by Regnase‐1 and Roquin is *Furin*, which encodes a conserved proprotein convertase crucial in human health and disease. Furin, along with other PCSK family members, is widely implicated in immune regulation, cancer and the entry, maturation or release of a broad array of evolutionarily diverse viruses including human papillomavirus (HPV), influenza (IAV), Ebola (EboV), dengue (DenV) and human immunodeficiency virus (HIV). Here, Braun and Sauter review the roles of furin in these processes, as well as the history and future of furin‐targeting therapeutics.^7^ They also discuss their recent work revealing how two IFN‐γ‐inducible factors exhibit broad‐spectrum inhibition of IAV, measles (MV), zika (ZikV) and HIV by suppressing furin activity.^8^ Over the coming decade, I expect to see an ever‐finer spatiotemporal resolution of host‐oriented therapies to achieve safe, effective and broad‐spectrum yet cost‐effective therapies for clinical use.

The increasing abundance of affordable, sensitive, high‐throughput genome sequencing technologies has led to a recent boom in metagenomics and the cataloguing of the microbiome of our world. The MinION nanopore sequencer is one of the latest innovations in this space, enabling direct sequencing in a miniature form factor with only minimal sample preparation and a consumer‐grade laptop computer. Nakagawa and colleagues here report on their latest experiments using this system, further improving its performance for use in resource‐poor contexts for meningitis diagnoses.^9^ While direct sequencing of viral genomic RNA is challenging, this system was recently used to directly sequence an RNA virus genome (IAV) for the first time.^10^ I anticipate further improvements in the performance of such devices over the coming decade will transform virus surveillance efforts, the importance of which was underscored by the recent EboV and novel coronavirus (nCoV / COVID‐19) outbreaks, enabling rapid deployment of antiviral treatments that take resistance‐conferring mutations into account.

Decades of basic immunology research have provided a near‐complete picture of the main armaments in the human antiviral arsenal. Nevertheless, this focus on mammalian defences and pathologies has sidelined examination of the types and roles of viruses and antiviral defences that exist throughout our biosphere. One case in point is the CRISPR/Cas antiviral immune system of prokaryotes, which is now repurposed as a revolutionary gene‐editing biotechnology in plants and animals.^11^ Another is the ancient lineage of nucleocytosolic large DNA viruses (NCLDVs), which are emerging human pathogens that possess enormous genomes of up to several megabases in size encoding hundreds of proteins with unique and unknown functions.^12^ Moreover, hundreds of human‐ and avian‐infective viruses such as IAV strain H5N1 are known, but recent efforts indicate the true number may be in the millions and many harbour zoonotic potential.^13^ It is increasingly clear that host–virus interactions have generated truly vast yet poorly understood and untapped biodiversity. Closing this Special Feature, Watanabe and Kawaoka elaborate on neo‐virology, an emerging field engaged in cataloguing and characterising this biodiversity through a global consortium.^14^ I predict these efforts will unlock a vast wealth of currently unexplored biodiversity, leading to biotechnologies and treatments that leverage the host–virus interactions developed throughout evolution.

When biomedical innovations fall into the ‘Valley of Death’, patients who are therefore not reached all too often fall with them. Being entrusted with the resources and expectation to conceive, deliver and communicate dividends to society is both cherished and eagerly pursued at every stage of our careers. Nevertheless, the road to research translation is winding and is built on a foundation of basic research. Supporting industry–academia collaboration and nurturing talent and skills in the Indo‐Pacific region are two of the four pillars of the National Innovation and Science Agenda.^2^ These frame Australia's Medical Research and Innovation Priorities, which include antimicrobial resistance, global health and health security, drug repurposing and translational research infrastructure,^15^ capturing many of the key elements of this CTI Special Feature. Establishing durable international relationships that integrate diverse expertise is essential to delivering these outcomes. To this end, NHMRC has recently taken steps under the International Engagement Strategy^16^ to increase cooperation with its counterparts overseas. These include the Japan Agency for Medical Research and Development (AMED), tasked with translating the biomedical research output of that country. Given the reciprocal efforts at accelerating bilateral engagement currently underway,^17^ the prospects for new areas of international cooperation and mobility have never been more exciting nor urgent. With the above in mind, all contributions to this CTI Special Feature I have selected from research presented by fellow invitees to the 2018 Awaji International Forum on Infection and Immunity (AIFII) and 2017 Consortium of Biological Sciences (ConBio) conferences in Japan. Both Australia and Japan have strong traditions in immunology and related disciplines, and I predict that the quantity, quality and importance of our bilateral cooperation will accelerate rapidly over the short to medium term. By expanding and cooperatively leveraging our respective research strengths, our efforts may yet solve the many pressing disease, cost and other sustainability issues of our time.

## Conflict of interest

The author declares no conflict of interest.
